# Supplementary material for: Splice-Junction-Based Mapping of Alternative Isoforms in the Human Proteome
Source: Cell Rep. Author manuscript; Available in PMC 2020 Jan 15. (PMC6961840; doi:10.1016/j.celrep.2019.11.026)
Supplement: 3 [file NIHMS1546469-supplement-3.zip › DF2/PXD0010154/Colon-31-Q15063-KLLQEDTPVRK.pdf]

A

Predicted sequence disorder and sequence features of Q15063

Peptide: KLLQEDTPVRK Junction: sp|Q15063|POSTN_HUMAN|ENSG00000133110|SE2|8269|chr13|37564560|37569383|−1|r7|T1 TrNovel: FALSE

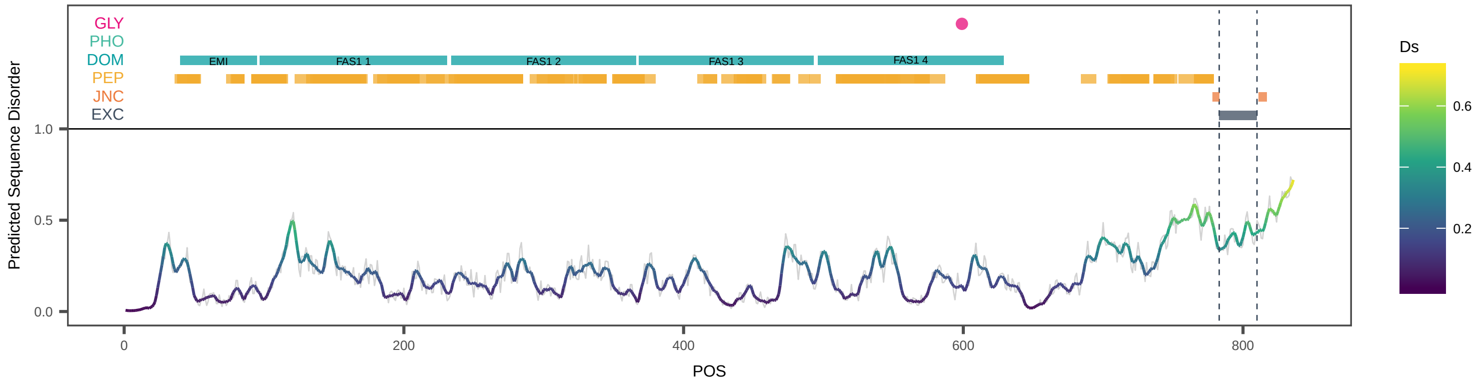

B

Distribution of sequence disorder in excised vs. mapped and non−excised regions of protein

M−W P−value vs. mapped: 1.03e−14 vs. non−excised: 2.05e−13

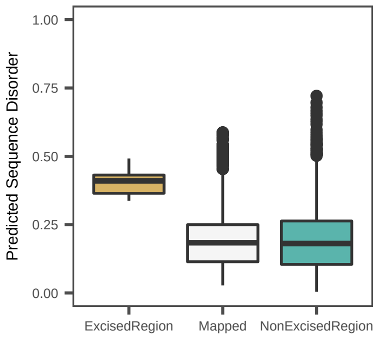

C
